# Trends in mortality after intensive care of patients with traumatic brain injury in Finland from 2003 to 2019: a Finnish Intensive Care Consortium study

**DOI:** 10.1007/s00701-021-05034-4

**Published:** 2021-11-02

**Authors:** Teemu Luostarinen, Juho Vehviläinen, Matias Lindfors, Matti Reinikainen, Stepani Bendel, Ruut Laitio, Sanna Hoppu, Tero Ala-Kokko, Markus Skrifvars, Rahul Raj

**Affiliations:** 1 Anaesthesiology and Intensive Care, Hyvinkää Hospital, Helsinki University Hospital and University of Helsinki, Helsinki, Finland; 2 Department of Neurosurgery, Helsinki University Hospital and University of Helsinki, Helsinki, Finland; 3 Department of Anaesthesiology and Intensive Care, Kuopio University Hospital & University of Eastern Finland, Kuopio, Finland; 4 Department of Perioperative Services, Intensive Care and Pain Management, Turku University Hospital & University of Turku, Turku, Finland; 5 Department of Intensive Care and Emergency Medicine Services, Tampere University Hospital & University of Tampere, Tampere, Finland; 6 Department of Intensive Care, Oulu University Hospital & University of Oulu, Oulu, Finland; 7 Department of Emergency Care and Services, University of Helsinki and Helsinki University Hospital, Helsinki, Finland

**Keywords:** Traumatic brain injury, Intensive care unit, Critical care, Mortality, Prognosis, Finland

## Abstract

**Background:**

Several studies have suggested no change in the outcome of patients with traumatic brain injury (TBI) treated in intensive care units (ICUs). This is mainly due to the shift in TBI epidemiology toward older and sicker patients. In Finland, the share of the population aged 65 years and over has increased the most in Europe during the last decade. We aimed to assess changes in 12-month and hospital mortality of patients with TBI treated in the ICU in Finland.

**Methods:**

We used a national benchmarking ICU database (Finnish Intensive Care Consortium) to study adult patients who had been treated for TBI in four tertiary ICUs in Finland during 2003–2019. We divided admission years into quartiles and used multivariable logistic regression analysis, adjusted for case-mix, to assess the association between admission year and mortality.

**Results:**

A total of 4535 patients were included. Between 2003–2007 and 2016–2019, the patient median age increased from 54 to 62 years, the share of patients having significant comorbidity increased from 8 to 11%, and patients being dependent on help in activities of daily living increased from 7 to 15%. Unadjusted hospital and 12-month mortality decreased from 18 and 31% to 10% and 23%, respectively. After adjusting for case-mix, a reduction in odds of 12-month and hospital mortality was seen in patients with severe TBI, intracranial pressure monitored patients, and mechanically ventilated patients. Despite a reduction in hospital mortality, 12-month mortality remained unchanged in patients aged ≥ 70 years.

**Conclusion:**

A change in the demographics of ICU-treated patients with TBI care is evident. The outcome of younger patients with severe TBI appears to improve, whereas long-term mortality of elderly patients with less severe TBI has not improved. This has ramifications for further efforts to improve TBI care, especially among the elderly.

**Supplementary Information:**

The online version contains supplementary material available at 10.1007/s00701-021-05034-4.

## Introduction

Traumatic brain injury (TBI) is a major cause of hospitalization and death worldwide, consequently causing a major socioeconomic burden [24]. Estimated mortality rates in Europe and the USA are 11.7/100,000 and 17/100,000, respectively [33]. The incidence of hospitalized TBI is estimated to be 262/100,000 [42]. Patients with the most severe TBIs are treated in intensive care units (ICUs). In Europe, the 6-month mortality of ICU-treated patients with TBI was as high as 40% in the 1980s/1990s [36, 37], but more recent studies have found it to be approximately 25% [50, 55].

Despite recent advances in multimodal monitoring, including intracranial pressure, partial brain tissue oxygenation, cerebral autoregulation monitoring, and microdialysis [13, 51], it remains controversial whether the outcomes of patients with TBI have actually improved [49]. Still, there are studies indicating improved survival [2, 20, 31].

The reason for unimproved outcomes has been speculated to be the changing epidemiology of patients with TBI toward older patients with more concomitant comorbidities [47]. Finland has one of the highest shares of the population aged 65 years and older, and the share of this age group has increased the most among European countries between 2010 and 2020 [16]. Thus, we aimed to assess changes in mortality after intensive care for TBI during a 17-year period using a large national ICU benchmarking database. We hypothesized that after adjusting for injury severity, mortality decreased with time despite the population getting older.

## Methods

### Study setting and population

We conducted a retrospective register-based study using the Finnish Intensive Care Consortium (FICC) database. The FICC database has previously been described in detail [46]. The FICC was established in 1994 as an ICU benchmarking project. Today, all ICUs in Finland, except one neurosurgical ICU, participate in the FICC. The database is maintained by TietoEVRY (Helsinki, Finland).

Neurosurgery and neurointensive care are provided at only five university hospitals in Finland. Four of these five units (in the university hospitals of Kuopio, Oulu, Tampere, and Turku) participate in the FICC. These hospitals cover approximately two-thirds of the Finnish population. From the FICC database, we extracted all patients admitted with a diagnosis indicating TBI between 2003 and 2019 in these four units. TBI was defined if the patient had an Acute Physiology and Chronic Health Evaluation (APACHE) III diagnosis indicating TBI with an International Classification of Diseases, 10th revision, diagnosis of S06.X.

We included adult patients (age ≥ 18 years) and excluded foreigners (data available for 2003–2013) and non-emergency admissions. We conducted full data analysis due to the low number of missing data.

### Definition of covariates and outcomes

We extracted all covariates from the FICC database. Age was defined upon admission. The Glasgow Coma Scale (GCS) score was defined as the worst measured GCS score during the first 24 h in the ICU, or the last reliable GCS score was used for intubated and/or sedated patients, according to the Simplified Acute Physiology Score (SAPS) II definition [19]. Preadmission functional status was defined as a modified version of the World Health Organization/Eastern Cooperative Oncology Group classification used by the FICC [38]. Significant comorbidity was recorded if one of the SAPS II or APACHE II comorbidities was present [19, 29]. ICP monitoring and mechanical ventilation values were obtained through Therapeutic Intervention Scoring System (TISS) 76 and TISS-28 recordings. In 2018, the FICC gradually changed from TISS-76 to the simpler TISS-28. The primary outcome was 12-month mortality, and the secondary outcome was hospital mortality. Data on mortality were obtained through the FICC database.

### Statistical analyses

For the statistical analyses, we used IBM SPSS Statistics 27 for Macintosh (IBM Corp., Armonk, NY, USA). We report categorical data as numbers with percentages. Categorical data were compared across groups using a two-sided *χ*^2^ test. We tested continuous data for normality using the Kolmogorov–Smirnov test. Normally distributed data are reported as means with standard deviations and were compared between groups using a *t*-test. Nonparametric data are reported as medians with interquartile ranges (IQR) and were compared across two groups using the Mann–Whitney U test and across several groups using the Kruskal–Wallis test.

To test the association between admission year and mortality, we used multivariable logistic regression. We adjusted for illness severity using age, sex, admission type, significant comorbidity, GCS score, modified SAPS II score (without age, GCS score, and admission type), and treating hospital [44]. We confirmed the model’s accuracy by assessing its discrimination (area under the receiver operating characteristic curve [AUROC]—ranging from 0.5 to 1.0, a higher value indicating better discrimination), calibration (Hosmer–Lemeshow *p*-test, *p*-value over 0.05 indicating good calibration), and explained variance (Nagelkerke *R*^2^—ranging from 0 to 1, a higher value indicating better-explained variance).

We grouped patients into equally sized quartiles as much as possible according to admission year (2003–2007, 2008–2011, 2012–2015, and 2016–2019). We conducted separate analyses for 12-month mortality and hospital mortality, as well as a second analysis, including preadmission functional status as a covariate (excluding 149 patients). In sensitivity analyses, we included admission year as a continuous variable. We performed subgroup analyses in predefined subgroups according to GCS score groups (3–8, 9–12, and 13–15), age groups (< 40 y, 41–69 y, and ≥ 70 y), mechanical ventilation status (yes/no), and ICP monitoring status (yes/no).

We followed the Strengthening the Reporting of Observational Studies in Epidemiology statement [15] for reporting results. The study was approved by the Finnish Institute for Health and Welfare (Dnro THL/1298/5.05.00/2019) and all participating university hospitals. There was no need for patient consent.

## Results

### Patient characteristics

**Fig. 1:**
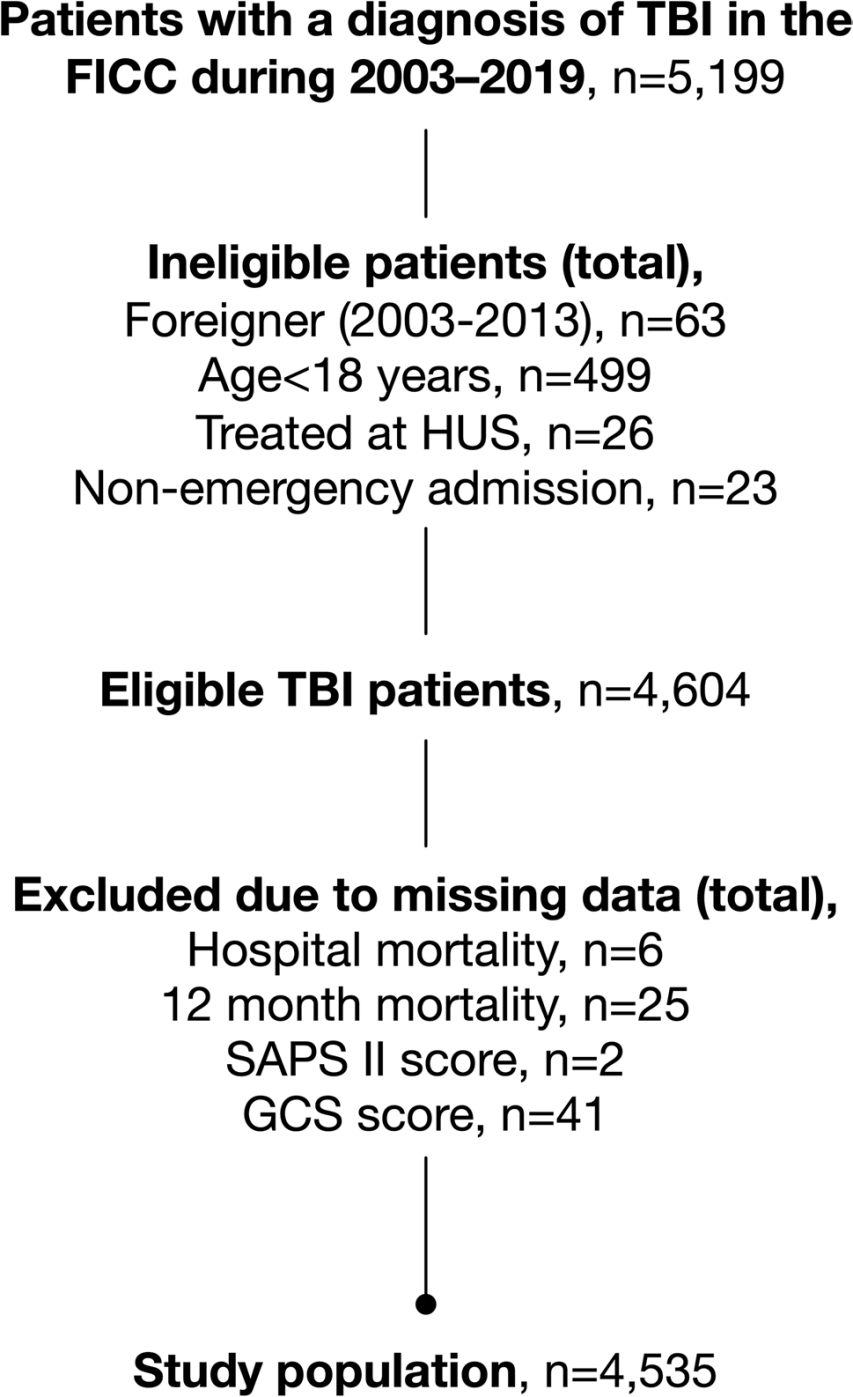
Flow chart showing study population

The study included 4535 patients treated in the ICU due to TBI (Fig. 1). The median age of the study population was 58 years (IQR 44–69). Seventy-six percent of the patients were male, 86% were functionally independent prior to admission, and 10% had significant comorbidity (Table 1). Almost two-thirds of the patients (63%) had a GCS score between 3 and 12. Thirty-one percent had an operative admission type, 23% were ICP monitored, and 61% were mechanically ventilated.

**Table 1:** Patient characteristics

Variable	All patients (*n* = 4535)
Age, median (IQR)	58 (44, 69)
< 40 y	919 (20%)
41–69 y	2536 (56%)
≥ 70 y	1080 (24%)
Sex	
Female	1107 (24%)
Male	3428 (76%)
Preadmission functional status^*^	
Independent	3911 (86%)
Dependent	475 (11%)
Significant comorbidity	430 (10%)
Operative admission	1399 (31%)
GCS score, median (IQR)	10 (5, 14)
3–8	2003 (44%)
9–12	887 (20%)
13–15	1645 (36.3%)
SAPS II score, median (IQR)	34 (23, 49)
ICP monitor^†^	1037 (23%)
Mechanical ventilation^†^	2770 (61%)
Mortality	
ICU	331 (7.3%)
Hospital	540 (12%)
12-month	1155 (26%)

^*^Missing for 149 patients

^†^Missing for 4 patients

*GCS*, Glasgow Coma Scale; *ICU*, intensive care unit; *ICP*, intracranial pressure; *IQR*, interquartile range; *SAPS*, simplified acute physiology score

The number of ICU-admitted patients with TBI increased from approximately 150–200 per year in the beginning to slightly over 300 patients per year toward the end (Supplementary eFigure 1). From 2003–2007 to 2016–2019, the median age increased (from 54 to 62 years, Table 2), the preadmission functional status “dependent on help in activities of daily living (ADL)” became more prevalent (from 7 to 15%), the presence of significant comorbidity became more prevalent (from 8 to 11%), operative admissions became less frequent (from 49 to 17%), GCS scores increased (proportion of patients with GCS 3–12 decreased from 79 to 54%), median SAPS II scores decreased (from 40 to 31), and the frequency of ICP monitoring (from 27 to 20%) and mechanical ventilation (from 75 to 53%) decreased. Median age increased similarly in all GCS groups (Supplementary eFigure 2). Furthermore, the increase in GCS score, the decrease in SAPS II score, and the decrease in operative admissions during the study period were consistent (Supplementary eFigures 3, 4, and 5).

**Table 2:** Patient characteristics according to time period

Variable	2003–2007 (*N* = 1068)	2008–2011 (*N* = 1081)	2012–2015 (*N* = 1225)	2016–2019 (*N* = 1161)	p-value
Age median (IQR)	54 (42, 65)	57 (44, 68)	59 (46, 70)	62 (45, 71)	< 0.001
< 40 y	236 (22%)	227 (21%)	228 (19%)	228 (20%)	< 0.001
41–69 y	648 (61%)	608 (56%)	683 (56%)	597 (52%)	
≥ 70 y	184 (17%)	246 (23%)	314 (26%)	336 (29%)	
Sex					0.099
Female	246 (23%)	243 (23%)	321 (26%)	297 (26%)	
Male	822 (77%)	838 (78%)	904 (74%)	864 (74%)	
Preadmission functional status^*^					< 0.001
Independent	962 (93%)	958 (90%)	1932 (89%)	959 (85%)	
Dependent	70 (7%)	107 (10%)	131 (11%)	167 (15%)	
Significant comorbidity	80 (8%)	98 (9%)	124 (10%)	128 (11%)	0.030
Operative admission	519 (49%)	410 (38%)	269 (22%)	201 (17%)	< 0.001
GCS score, median (IQR)	7 (4, 12)	9 (5, 13)	11 (6, 14)	12 (6, 14)	< 0.001
3–8	633 (59%)	487 (45%)	465 (38%)	418 (36%)	
9–12	209 (20%)	242 (22%)	233 (19%)	203 (18%)	
13–15	226 (21%)	352 (33%)	527 (43%)	540 (47%)	
SAPS II score, median (IQR)	40 (27, 53)	34 (24, 49)	31 (22, 47)	31 (21, 45)	< 0.001
Modified SAPS II, median (IQR)	10 (5, 15)	8 (4, 13)	8 (4, 13)	7 (3, 13)	< 0.001
ICP monitor ^†^	283 (27%)	244 (23%)	274 (22%)	236 (20%)	0.006
Mechanical ventilation^†^	805 (75%)	685 (63%)	671 (55%)	609 (53%)	< 0.001
Mortality					
ICU	115 (11%)	65 (6%)	73 (6%)	78 (7%)	< 0.001
Hospital	190 (18%)	128 (12%)	109 (9%)	113 (10%)	< 0.001
12-month	333 (31%)	283 (26%)	267 (22%)	272 (23%)	< 0.001

^*^Missing for 149 patients

^†^Missing for 4 patients

^‡^Tested between nonparametric variables using the independent samples Kruskal–Wallis test and between categorical variables using a two-sided unadjusted chi-square test

*GCS*, Glasgow Coma Scale; *ICU*, intensive care unit; *ICP*, intracranial pressure; *IQR*, interquartile range; *SAPS*, simplified acute physiology score

### Association between admission year and mortality

Twelve-month mortality was 1155/4535 (26%), out of which 540/1155 (47%) occurred during the index hospitalization. The unadjusted 12-month mortality decreased from 31.2 to 23.4% between 2003–2007 and 2016–2019. The unadjusted hospital mortality decreased from 17.8 to 9.7% between 2003–2007 and 2016–2019.

The AUROC for the illness severity model predicting 12-month mortality was 0.85 (95% CI 0.84–0.86), the Hosmer–Lemeshow *p*-value was 0.264, and Nagelkerke *R*^2^ was 0.42. For predicting hospital mortality, the AUROC for the illness severity model was 0.91 (95% CI 0.90–0.92), the Hosmer–Lemeshow *p*-value was < 0.001, and Nagelkerke *R*^2^ was 0.50.

**Fig. 2:**
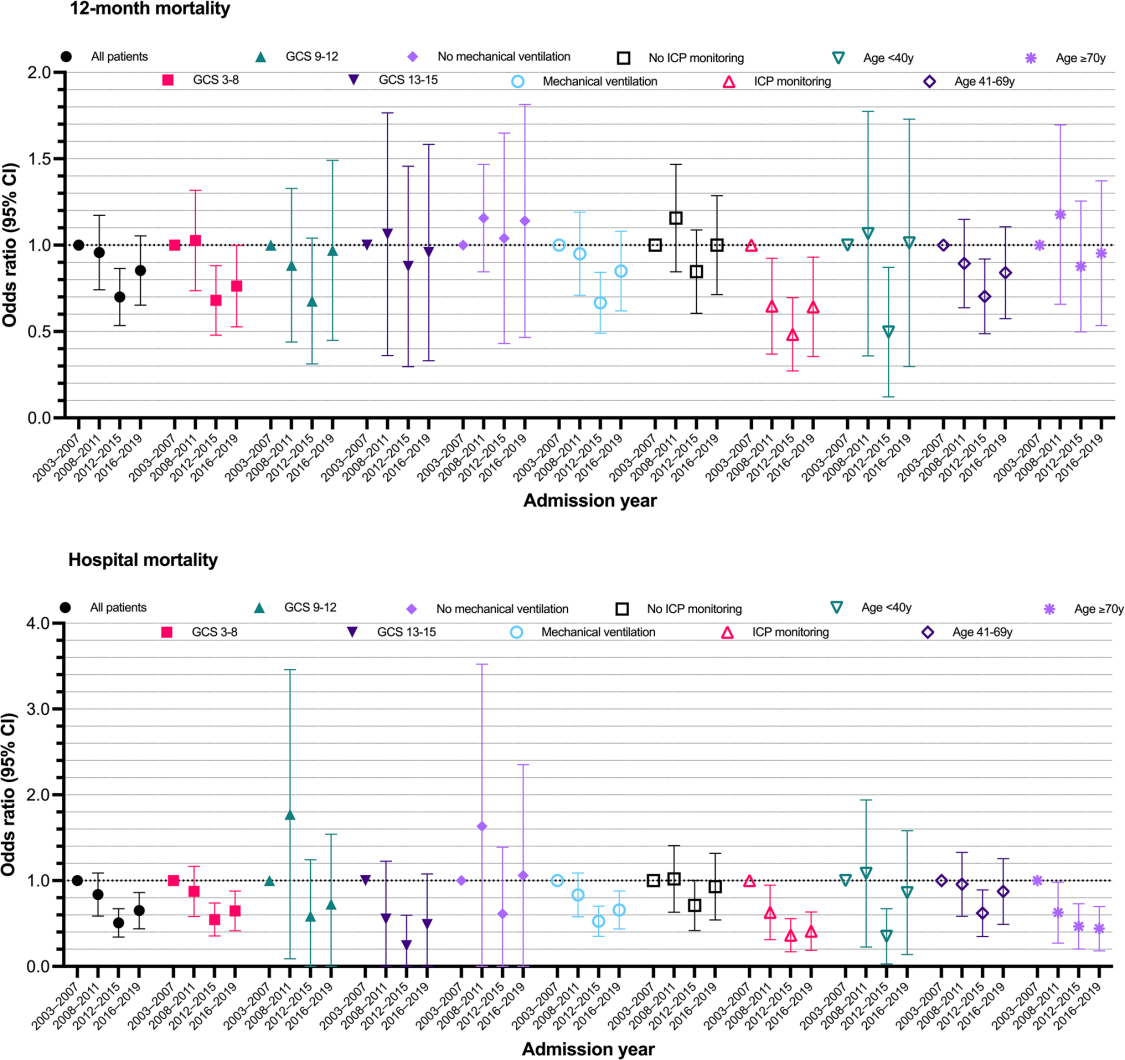
Showing the odds ratio (with 95% CI) for adjusted 12-month mortality (upper panel) and adjusted hospital mortality (lower panel) for all patients and predefined subgroups after adjusting for injury severity (age, gender, GCS score, significant comorbidity, modified SAPS II, treatment hospital) using multivariable logistic regression. A reduction in adjusted 12-month mortality was seen in patients with a GCS score of 3–8, in patients being mechanically ventilated and undergoing ICP monitoring. A reduction in adjusted hospital mortality was seen in patients with a GCS score 3–8, mechanically ventilated patients, ICP monitored patients, and in patients 70 years or older. Patients with a GCS of 13–15 seemed to have lower odds for hospital mortality, but this subgroup suffered from a low number of events (only 14 out of 1645 patients died). *CI*, confidence intervals; *GCS*, Glasgow Coma Scale

Including all patients, a clear trend toward reduced odds of 12-month mortality and hospital mortality as a function of time was seen (Table 3). The reduction in odds for death was more pronounced for hospital mortality compared to 12-month mortality (Fig. 2). In a second analysis, including preadmission functional status (missing for 149 patients), the results remained the same (Supplementary eTable 1). Patients being dependent on help in ADL had 1.5 higher odds for 12-month mortality but no increased odds for hospital mortality.

**Table 3:** Results from the multivariable logistic regression analysis

Variable	OR (95% CI)	p-value
	12-month mortality	
Age	1.05 (1.05–1.06)	< 0.01^*^
Female	0.85 (0.70–1.02)	0.09
GCS score	0.79 (0.78–0.81)	< 0.01^*^
Significant comorbidity	2.35 (1.82–3.03)	< 0.01^*^
Operative admission	0.76 (0.64–0.92)	< 0.01^*^
Modified SAPS II	1.08 (1.07–1.10)	< 0.01^*^
Admission year		
2003–2007	Ref	
2008–2011	0.94 (0.75–1.18)	0.58
2012–2015	0.69 (0.54–0.87)	< 0.01^*^
2016–2019	0.84 (0.66–1.06)	0.14
	Hospital mortality	
Age	1.03 (1.02–1.03)	< 0.01^*^
Female	0.90 (0.69–1.18)	0.45
GCS score	0.66 (0.63–0.69)	< 0.01^*^
Operative admission	0.54 (0.42–0.70)	< 0.01^*^
Significant comorbidity	1.54 (1.09–2.19)	0.02^*^
Modified SAPS II	1.23 (1.11–1.14)	< 0.01^*^
Admission year		
2003–2007	Ref	
2008–2011	0.81 (0.60–1.10)	0.18
2012–2015	0.49 (0.35–0.68)	< 0.01^*^
2016–2019	0.63 (0.45–0.87)	< 0.01^*^

All models adjusted for treatment hospital

^*^p < 0.05

*GCS*, Glasgow Coma Scale; *SAPS*, Simplified Acute Physiology Score

Subgroup analysis showed a trend toward lower odds of 12-month mortality in the GCS 3–8 group, mechanically ventilated patients, and ICP-monitored patients (Fig. 2, Supplementary eTable 2). Regarding hospital mortality, there was a trend toward lower odds of death in GCS 3–8, GCS 13–15 (only 14 out of 1645 died), mechanically ventilated, and ICP-monitored patients, as well as in patients ≥ 70 years (Fig. 2).

In the sensitivity analyses, using admission year as a continuous variable, a consistently lower odds of 12-month mortality as a function of time was seen in patients with GCS 3–8 and in those ICP monitored (Supplementary eTable 3). Regarding hospital mortality, a consistently lower odds for hospital mortality was seen in patients with GCS 3–8, mechanically ventilated and ICP-monitored patients, and patients ≥ 70 years.

## Discussion

### Key findings

In this large multicenter observational study, including 4535 patients from four academic centers in Finland, we showed that both hospital mortality and 12-month mortality decreased during 2003–2019 for patients with TBI being treated in the ICU. The reduction in mortality was mainly due to improved survival in patients with the most severe TBIs (GCS 3–8, ICP monitored, mechanically ventilated). Furthermore, a reduction in the odds of hospital mortality was seen in patients 70 years and older, although the odds for 12-month mortality were unchanged.

### Epidemiology of intensive care unit–treated patients with traumatic brain injury

The epidemiology and cause of TBI and patient characteristics have changed during the past decades [47]. In most high-income countries, the rate of high-energy motor vehicle accidents is decreasing and that of low-energy falling accidents is increasing [1, 25, 53, 57]. This is a natural consequence of the aging population being more prone to falling accidents. In parallel with this, improvements in road infrastructure, car safety, and legislation have markedly reduced the number of road traffic deaths from 379 in 2003 (total population: 5.2 million) to 211 in 2019 in Finland (total population: 5.5 million, data from Statistics Finland [https://pxnet2.stat.fi/PXWeb/pxweb/fi/StatFin/]). The epidemiological shift is well demonstrated in our study by increased age, more concomitant comorbidities, poorer pretrauma functional status, lower GCS scores, lower SAPS II scores, and a reduced need for operations, ICP monitoring, and mechanical ventilation. For example, a similar decline in the need for operative treatment has been reported in North America [18]. Of note, the temporal change in treatment patterns is probably just a reflection of the shift in patient demography and of the increased availability of ICU beds over time. Thus, it is likely that more elderly patients with reduced pretrauma functional capacity are now being admitted to the ICU because of the increased availability of ICU beds.

The increased share of older patients, patients being dependent on ADL, and patients having significant comorbidities poses major challenges. We found that although hospital mortality decreased in patients aged 70 years and over, possibly indicating improved intensive care or more restricted admission criteria, 12-month mortality did not change. Thus, there is a clear need to establish whether intensive care for elderly patients with TBI is justified.

### Outcome after intensive care of patients with traumatic brain injury

Regarding the outcome of TBI in general, a thorough review covering the last 150 years reported no improvement in mortality since 1990, after two decades of a marked decrease in mortality rates [49]. More recent reports, however, indicate that the outcome of patients with TBI may be improving [20, 26, 27, 31, 44].

Although there has been no single randomized controlled trial showing a benefit in terms of patient outcome regarding a single therapeutic agent or intervention (decompressive craniectomy [23] and tranexamic acid [12] being debated), one can argue that, overall, neurointensive and neurosurgical care improved during the study period. Up-to-date treatment guidelines published in 1997 [32], 2007 [6], and 2017 [8] were widely adopted in Finland. Although the guidelines are partly based on consensus statements and partly on evidence, adherence to treatment guidelines has been shown to improve outcomes by standardizing care [2, 17]. Improved adherence to the guidelines over time (further improving the quality of care) is likely to have occurred, although we cannot confirm this. However, the fact that mortality decreased in those patients portrayed in the guidelines (GCS 3–8 and ICP-monitored patients) and not in patients not portrayed in the guidelines (e.g., patients with GCS 13–15) supports this hypothesis. Furthermore, the presence and continued education of dedicated intensivists, neurosurgeons, and intensive care nurses is likely to have contributed to the improved outcome.

One should acknowledge that TBI care is a chain that includes prehospital care, ICU care, and rehabilitation. Developments in prehospital care, such as rapid sequence intubation [5], the presence of on-scene emergency physicians [40], and rehabilitation (such as early and intensive neurorehabilitation) [30], are likely to contribute to the improvements noted. In addition, changes in patient selection (e.g., decompressive craniectomy candidates [11, 23]) may have had an impact on the outcome of our study.

New promises in goal-directed ICU management of TBI include brain tissue oxygen monitoring and treatment [39], optimal cerebral perfusion pressure targeting [4], brain tissue microdialysis [54], spreading depolarization monitoring and treatment [22], and a combination of these into a multimodal monitoring approach [28]. However, all these still lack level 1 evidence. The latest addition to multimodal neuromonitoring of patients with TBI is artificial intelligence [45].

### Comparison to previous studies

Our previous report from the one neurosurgical ICU not participating in the FICC showed a significant reduction in odds for an unfavorable functional outcome but no change in mortality [31]. In addition, studies from North America [20], the United Kingdom [41], Italy [14], South Korea [27], Canada [48], Austria [35], and Japan [56] have shown a reduction in mortality over time.

However, there are studies from Australia [3], Iceland [25], Canada [7], Greece [52], and Belgium [43] that do not show any change in outcome after TBI. Importantly, mortality in China [9] and India [34], where the incidence of TBI [24] is the highest, has not markedly changed.

Based on these findings, it appears that mortality has decreased in most high-income countries, whereas in countries with poorer infrastructure and more road traffic accidents, mortality has remained unchanged. Consequently, the reduction in mortality after TBI might just reflect safer roads in these high-income countries instead of being a marker of improved health care. Moreover, it is possible that the implementation of treatment guidelines and the capacity for multimodal neuromonitoring, for instance, are not as widespread in low-income countries as they are in high-income ones.

### Strengths and limitations

The major strengths of the present study are the long study period, the large sample size, and the use of high-quality data. The FICC is a high-quality, multicenter ICU database that prospectively collects data for all patients treated in the participating ICUs. For example, before entering data into the FICC, specially trained personnel verify the quality and consistency of the data. In Finland, tertiary care of TBI has, for decades, been centralized to the university hospitals. Four out of five university hospitals treating patients with TBI participate in the FICC, enabling us to analyze two-thirds of the Finnish population.

Some limitations should be mentioned. First, as the FICC is a general ICU database, it lacks some TBI-specific parameters, such as admission GCS score, pupillary reactivity, CT findings, and specific neurosurgical procedures. Still, for case-mix adjustment, our model displayed good statistical performance. Second, we could not control for prehospital care or rehabilitation. Substantial variations in prehospital care and rehabilitation for TBI exist in Europe [10, 21]. It is likely that these variations are smaller within Finland but cannot be excluded. Third, we only had all-cause mortality as an outcome measure. Functional outcome, quality of life, and social and neuropsychological outcomes are crucial, and modern ICU databases should start collecting these routinely. Fourth, we highlight that we only included university hospital–treated TBIs and did not include milder TBIs treated at local central hospitals or out-of-hospital TBI deaths. Fifth, although all centers adhered to the most up-to-date Brain Trauma Foundation guidelines, we could not control for guideline adherence nor how specific changes in prehospital care, in-ICU care, or post-ICU care affected patient outcomes.

## Conclusion

In Finland, a decrease in the prevalence of ICU-admitted patients with severe TBI, patients with TBI needing ICP monitoring and mechanical ventilation, was seen. However, the risk of 12-month mortality seemed to decrease among patients with severe TBI being ICP monitored and mechanically ventilated despite the change in patient epidemiology toward older and sicker patients. The outcome of younger patients with severe TBI appears to improve, whereas the long-term outcome of elderly patients with less severe TBI has not. There is an evident need to establish the benefits of intensive care among elderly patients with TBI.

## Supplementary Information

Below is the link to the electronic supplementary material.

Supplementary file1 (DOCX 765 KB)

## Data Availability

The datasets analyzed during the current study are not publicly available due to restrictions based on the General Data Protection Regulation (GDPR) on sensitive data such as personal health data. Access to the data may be requested through the Finnish Institute for Health and Welfare (THL) Biobank (https://thl.fi/en/web/thl-biobank/for-researchers).
