# Supplementary material for: Trends in mortality after intensive care of patients with traumatic brain injury in Finland from 2003 to 2019: a Finnish Intensive Care Consortium study
Source: Acta Neurochir (Wien). 2021 Nov 2;164(1):87–96. doi: 10.1007/s00701-021-05034-4 (PMC8761133; doi:10.1007/s00701-021-05034-4)
Supplement: Supplementary file 1 — Supplementary file1 (DOCX 765 KB) [file 701_2021_5034_MOESM1_ESM.docx]

| **eTable 1**: Results from the multivariable logistic regression analysis including preadmission functional status | | |
| --- | --- | --- |
| **Variable** | **OR (95% CI)** | **p-value** |
| **12-month mortality** | | |
| **Age** | 1.05 (1.05 - 1.06) | <0.01* |
| **Female** | 0.82 (0.68 - 1.00) | 0.05 |
| **GCS score** | 0.79 (0.77 - 0.81) | <0.01* |
| **Significant comorbidity** | 2.30 (1.78 - 2.97) | <0.01* |
| **Operative admission** | 0.75 (0.62 - 0.90) | <0.01* |
| **Modified SAPS II** | 1.08 (1.07 - 1.10) | <0.01* |
| **Preadmission functional status** |  |  |
| Independent in ADL | Ref |  |
| Dependent in ADL | 1.50 (1.14 - 1.90) | <0.01* |
| **Admission year** |  |  |
| 2003–2007 | Ref |  |
| 2008–2011 | 0.92 (0.73 - 1.17) | 0.48 |
| 2012–2015 | 0.68 (0.53 - 0.86) | <0.01* |
| 2016–2019 | 0.82 (0.64 - 1.05) | 0.11 |
| **Hospital mortality** | | |
| **Age** | 1.03 (1.02 - 1.04) | <0.01* |
| **Female** | 0.86 (0.66 - 1.14) | 0.30 |
| **GCS score** | 0.66 (0.63 - 0.69) | <0.01* |
| **Operative admission** | 0.55 (0.42 - 0.71) | <0.01* |
| **Significant comorbidity** | 1.62 (1.14 - 2.30) | <0.01* |
| **Modified SAPS II** | 1.12 (1.10 - 1.14) | <0.01* |
| **Preadmission functional status** |  |  |
| Independent in ADL | Ref |  |
| Dependent in ADL | 0.78 (0.53 - 1.13) | 0.78 |
| **Admission year** |  |  |
| 2003–2007 | Ref |  |
| 2008–2011 | 0.80 (0.59 - 1.08) | 0.15 |
| 2012–2015 | 0.47 (0.34 - 0.66) | <0.01* |
| 2016–2019 | 0.62 (0.44 - 0.86) | <0.01* |
| All models adjusted for treatment hospital  *p<0.05  Abbreviations: ADL=Activities of Daily Living, GCS=Glasgow Coma Scale, SAPS=Simplified Acute Physiology Score. | | |

| **eTable 2**: Results from the multivariable logistic regression analysis for predefined subgroups | | |
| --- | --- | --- |
| **Subgroup** | **Admission year OR (95% CI)** | **p-value** |
| **12-month mortality** | | |
| **GCS 3–8** |  |  |
| 2003–2007 | 1.0 |  |
| 2008–2011 | 1.00 (0.75 - 1.33) | 0.98 |
| 2012–2015 | 0.66 (0.49 - 0.89) | <0.01* |
| 2016–2019 | 0.74 (0.54 - 1.01) | 0.06 |
| **GCS 9–12** |  |  |
| 2003–2007 | 1.0 |  |
| 2008–2011 | 0.81 (0.48 - 1.36) | 0.42 |
| 2012–2015 | 0.61 (0.35 - 1.07) | 0.09 |
| 2016–2019 | 0.88 (0.50 - 1.53) | 0.65 |
| **GCS 13–15** |  |  |
| 2003–2007 | 1.0 |  |
| 2008–2011 | 0.91 (0.45 - 1.83) | 0.78 |
| 2012–2015 | 0.75 (0.37 - 1.51) | 0.42 |
| 2016–2019 | 0.82 (0.41 - 1.64) | 0.57 |
| **Age <40y** |  |  |
| 2003–2007 | 1.0 |  |
| 2008–2011 | 0.91 (0.45–1.84) | 0.79 |
| 2012–2015 | 0.40 (0.18–0.91) | 0.03* |
| 2016–2019 | 0.84 (0.40–1.80) | 0.66 |
| **Age 41–69y** |  |  |
| 2003–2007 | 1.0 |  |
| 2008–2011 | 0.87 (0.65–1.16) | 0.33 |
| 2012–2015 | 0.68 (0.50–0.93) | 0.02* |
| 2016–2019 | 0.81 (0.59–1.12) | 0.19 |
| **Age ≥70y** |  |  |
| 2003–2007 | 1.0 |  |
| 2008–2011 | 1.10 (0.70–1.73) | 0.68 |
| 2012–2015 | 0.82 (0.53–1.28) | 0.38 |
| 2016–2019 | 0.89 (0.57–1.40) | 0.62 |
| **No mechanical ventilation** |  |  |
| 2003–2007 | 1.0 |  |
| 2008–2011 | 1.13 (0.62 - 2.06) | 0.69 |
| 2012–2015 | 0.92 (0.50 - 1.70) | 0.79 |
| 2016–2019 | 1.01 (0.54 - 1.87) | 0.98 |
| **Mechanical ventilation** |  |  |
| 2003–2007 | 1.0 |  |
| 2008–2011 | 0.93 (0.72 - 1.20) | 0.57 |
| 2012–2015 | 0.65 (0.50 - 0.85) | <0.01* |
| 2016–2019 | 0.83 (0.63 - 1.09) | 0.17 |
| **No ICP monitor** |  |  |
| 2003–2007 | 1.0 |  |
| 2008–2011 | 1.13 (0.86 - 1.48) | 0.39 |
| 2012–2015 | 0.82 (0.62 - 1.10) | 0.19 |
| 2016–2019 | 0.97 (0.73 - 1.30) | 0.86 |
| **ICP monitor** |  |  |
| 2003–2007 | 1.0 |  |
| 2008–2011 | 0.61 (0.39 - 0.94) | 0.03* |
| 2012–2015 | 0.45 (0.29 - 0.71) | <0.01* |
| 2016–2019 | 0.60 (0.38 - 0.95) | 0.03* |
| **Hospital mortality** | | |
| **GCS 3–8** |  |  |
| 2003–2007 | 1.0 |  |
| 2008–2011 | 0.84 (0.60 - 1.18) | 0.31 |
| 2012–2015 | 0.52 (0.37 - 0.75) | <0.01* |
| 2016–2019 | 0.62 (0.43 - 0.89) | <0.01* |
| **GCS 9–12** |  |  |
| 2003–2007 | 1.0 |  |
| 2008–2011 | 1.24 (0.42 - 3.66) | 0.70 |
| 2012–2015 | 0.34 (0.085 - 1.33) | 0.12 |
| 2016–2019 | 0.42 (0.11 - 1.65) | 0.21 |
| **GCS 13–15** |  |  |
| 2003–2007 | 1.0 |  |
| 2008–2011 | 0.28 (0.057 - 1.32) | 0.11 |
| 2012–2015 | 0.067 (0.007 - 0.65) | 0.02* |
| 2016–2019 | 0.25 (0.055 - 1.16) | 0.08 |
| **Age <40y** |  |  |
| 2003–2007 | 1.0 |  |
| 2008–2011 | 0.86 (0.36–2.03) | 0.72 |
| 2012–2015 | 0.25 (0.09–0.71) | 0.01* |
| 2016–2019 | 0.66 (0.26–1.66) | 0.38 |
| **Age 41–69y** |  |  |
| 2003–2007 | 1.0 |  |
| 2008–2011 | 0.91 (0.61–1.35) | 0.63 |
| 2012–2015 | 0.58 (0.37–0.91) | 0.02* |
| 2016–2019 | 0.82 (0.52–1.28) | 0.38 |
| **Age ≥70y** |  |  |
| 2003–2007 | 1.0 |  |
| 2008–2011 | 0.56 (0.31–1.01) | 0.06 |
| 2012–2015 | 0.42 (0.23–0.75) | <0.01* |
| 2016–2019 | 0.39 (0.21–0.72) | <0.01* |
| **No mechanical ventilation** |  |  |
| 2003–2007 | 1.0 |  |
| 2008–2011 | 0.90 (0.22 - 3.78) | 0.89 |
| 2012–2015 | 0.28 (0.054 - 1.50) | 0.14 |
| 2016–2019 | 0.54 (0.11 - 2.53) | 0.43 |
| **Mechanical ventilation** |  |  |
| 2003–2007 | 1.0 |  |
| 2008–2011 | 0.81 (0.59 - 1.10) | 0.18 |
| 2012–2015 | 0.51 (0.36 - 0.71) | <0.01* |
| 2016–2019 | 0.63 (0.45 - 0.89) | <0.01* |
| **No ICP monitor** |  |  |
| 2003–2007 | 1.0 |  |
| 2008–2011 | 0.97 (0.66 - 1.43) | 0.87 |
| 2012–2015 | 0.67 (0.44 - 1.02) | 0.06 |
| 2016–2019 | 0.88 (0.57 - 1.34) | 0.54 |
| **ICP monitor** |  |  |
| 2003–2007 | 1.0 |  |
| 2008–2011 | 0.58 (0.34 - 0.97) | 0.04* |
| 2012–2015 | 0.33 (0.19 - 0.57) | <0.01* |
| 2016–2019 | 0.37 (0.21 - 0.65) | <0.01* |
| Model adjusted for age, gender, GCS score, significant comorbidity, modified SAPS II and treatment hospital.  *p<0.05  Abbreviations: GCS=Glasgow Coma Scale, ICP=Simplified Acute Physiology Score II. | | |

| **eTable 3**: Multivariable logistic regression model for odds of death using admission year as a continuous variable | | |
| --- | --- | --- |
| **Subgroup** | **Admission year OR (95% CI)** | **p-value** |
| **12-month mortality** | | |
| All patients | 0.98 (0.97–1.00) | 0.10 |
| GCS 3–8 | 0.97 (0.95–0.996) | 0.02* |
| GCS 9–12 | 1.00 (0.96–1.04) | 0.94 |
| GCS 13–15 | 0.99 (0.94–1.04) | 0.69 |
| No mechanical ventilation | 1.00 (0.96–1.06) | 0.73 |
| Mechanical ventilation | 0.98 (0.96–1.00) | 0.07 |
| No ICP monitoring | 1.00 (0.97–1.02) | 0.65 |
| ICP monitoring | 0.96 (0.93–0.99) | 0.02* |
| Age <40y | 0.96 (0.91–1.02) | 0.23 |
| Age 41–69y | 0.98 (0.96–1.01) | 0.15 |
| Age ≥70y | 0.99 (0.96–1.03) | 0.67 |
| **Hospital mortality** | | |
| All patients | 0.96 (0.93–0.98) | <0.01* |
| GCS 3–8 | 0.95 (0.93–0.98) | <0.01* |
| GCS 9–12 | 0.93 (0.84–1.03) | 0.93 |
| GCS 13–15 | 0.90 (0.78–1.03) | 0.90 |
| No mechanical ventilation | 0.93 (0.83–1.05) | 0.23 |
| Mechanical ventilation | 0.96 (0.93–0.98) | <0.01* |
| No ICP monitoring | 0.98 (0.95–1.01) | 0.27 |
| ICP monitoring | 0.91 (0.87–0.96) | <0.01* |
| Age <40y | 0.93 (0.86–0.997) | 0.04* |
| Age 41–69y | 0.98 (0.64–1.01) | 0.19 |
| Age ≥70y | 0.93 (0.89–0.98) | <0.01* |
| Model adjusted for age, gender, GCS score, significant comorbidity, modified SAPS II and treatment hospital.  *p<0.05  Abbreviations: GCS=Glasgow Coma Scale, ICP=Simplified Acute Physiology Score II. | | |

**eFigure 1**


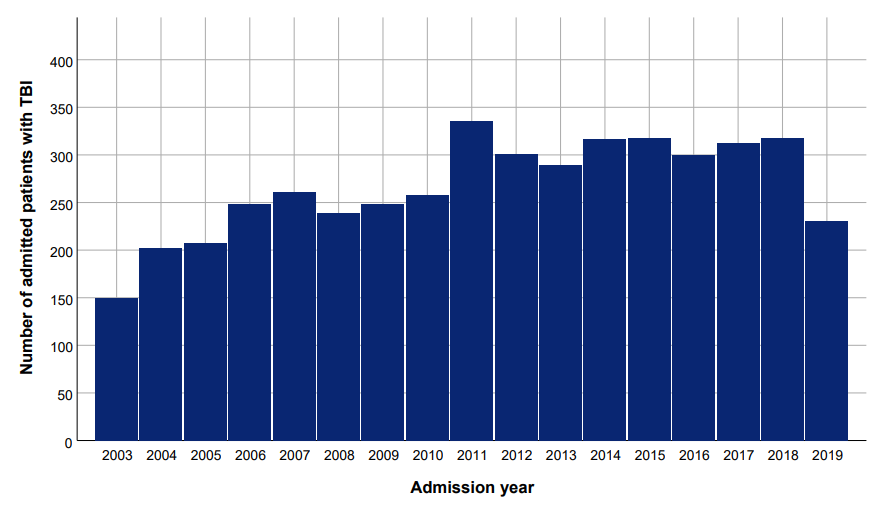


**eFigure 1**: The number of intensive care unit admitted patients with TBI increased consistently during the study period.

**eFigure 2**


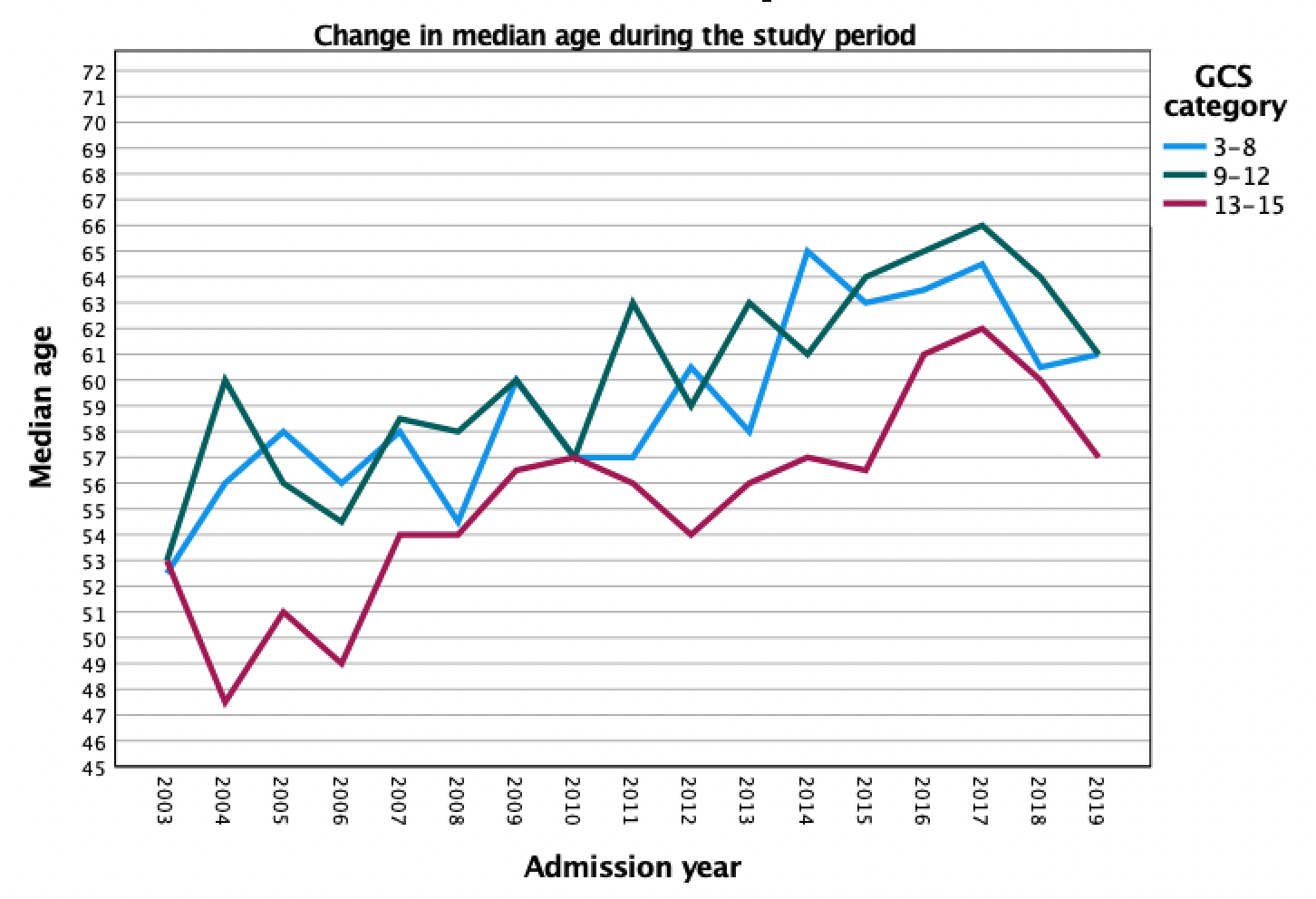


**eFigure 2**: Change in median age according to Glasgow Coma Scale score category during 2003–2019

**eFigure 3**


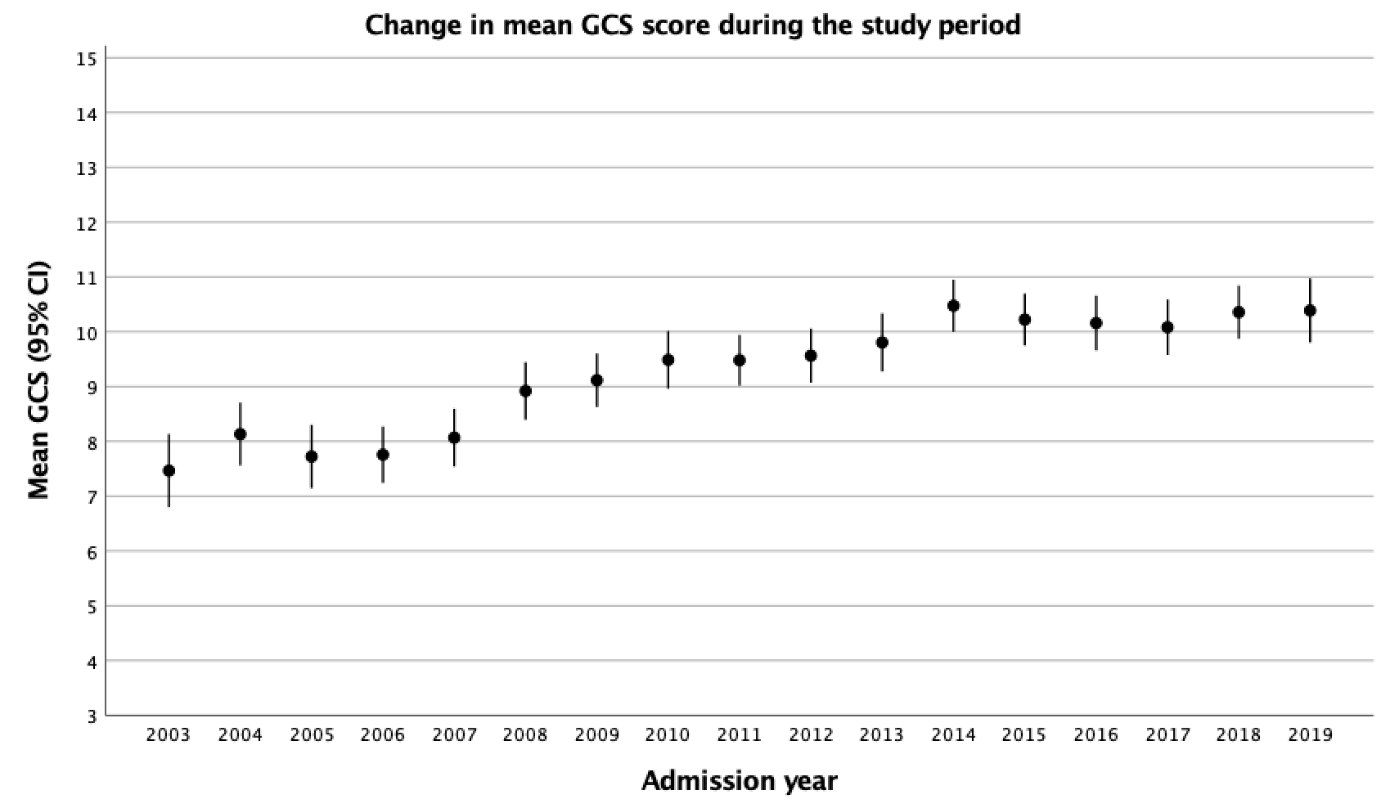


**eFigure 3:** Change in mean Glasgow Coma Scale (GCS) score (with 95% confidence intervals) during 2003–2019. The GCS score is defined as the worst measured GCS score during the first ICU day or the last reliable GCS was used for intubated and/or sedated patients according to the SAPS II definition.

**eFigure 4**


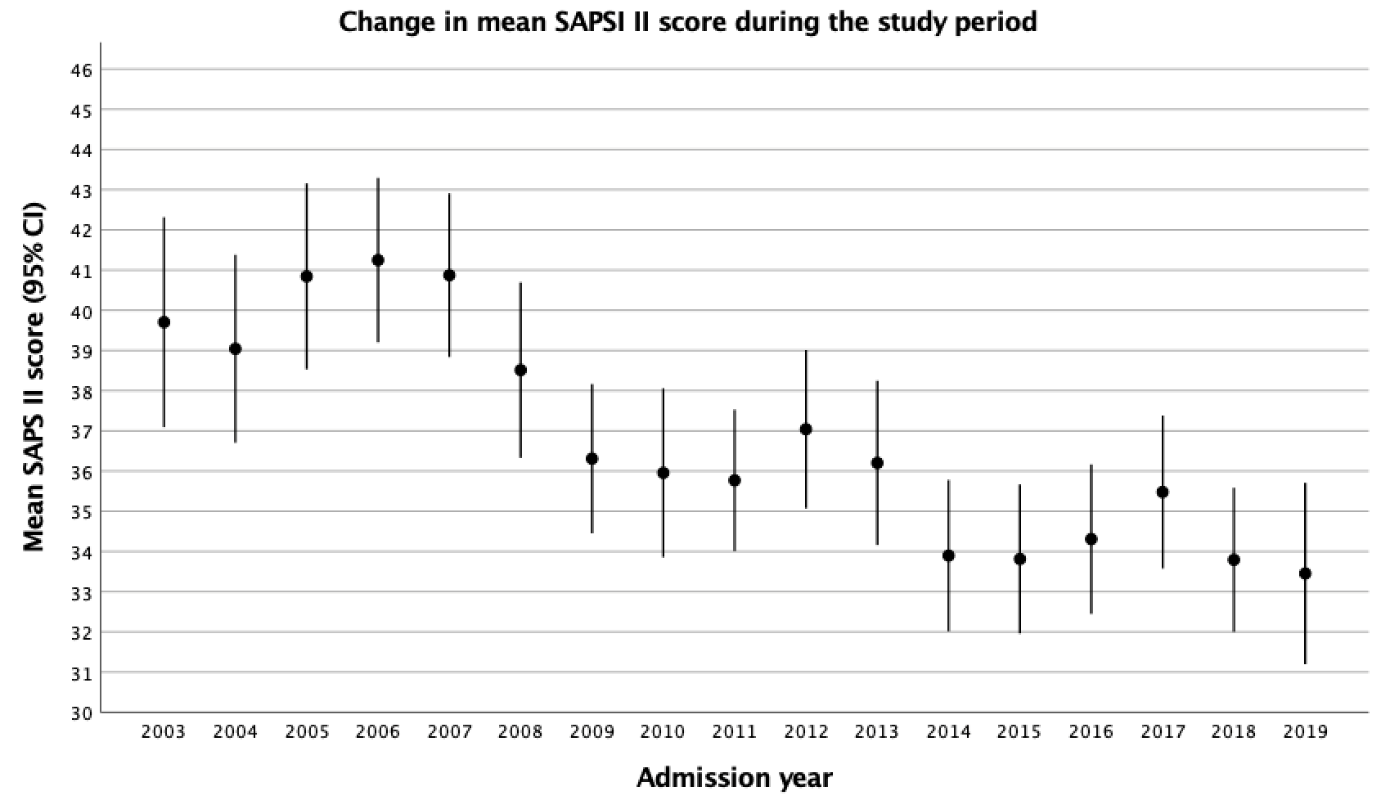


**eFigure 4:** Change in mean SAPS II (Simplified Acute Physiology Score II) during the study period

**eFigure 5**

**
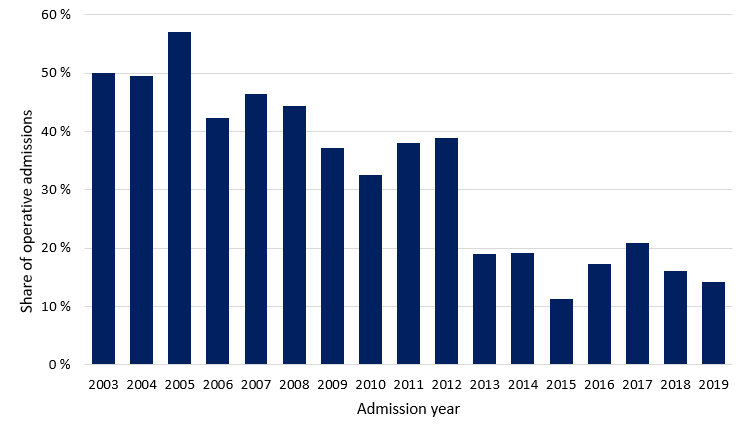
**

**eFigure 5**: The share of operative admissions decreased consistently during the study period from 50% in 2003 to 14% in 2019.
